# Germ cell‐specific expression of Cre recombinase using the *VASA* promoter in the pig

**DOI:** 10.1002/2211-5463.12005

**Published:** 2015-12-29

**Authors:** Yuning Song, Liangxue Lai, Li Li, Yongye Huang, Anfeng Wang, Xiaochun Tang, Daxin Pang, Zhanjun Li, Hongsheng Ouyang

**Affiliations:** ^1^ Jilin Provincial Key Laboratory of Animal Embryo EngineeringJilin UniversityChangchunChina

**Keywords:** Cre–*lox*P system, pig, SCNT, transgene, *VASA*

## Abstract

The Cre–*lox*P system is a powerful tool for genetic analysis of distinct cell lineages and tissue‐specific gene knockout in animal models. *VASA* is specifically expressed in reproductive tissues, and is known to play important roles in spermatogenesis and germ‐cell growth. In this study, Cre recombinase transgenic pigs under the control of the *VASA* promoter were generated by somatic cell nuclear transfer. Germ cell‐specific expression of Cre recombinase in *VASA*‐Cre transgenic pigs was shown by western blotting and immunohistochemistry. *VASA*‐Cre transgenic pigs will be a useful tool for germ cell‐specific gene knockout and a disease model for disorders of the reproductive system.

**Abbreviations**
293T: human kidney epithelial cell line
HE: haematoxylin–eosin
IHC: immunohistochemistry
MLTC‐1: mouse Leydig tumour cell line
PEF: porcine fetal fibroblast cell line
PK: pig kidney epithelial cell line
SCNT: somatic cell nuclear transfer
Tg: transgenic
WT: wild‐type

The Cre–*lox*P system has been widely used for spatial and temporal deletion of genes in yeast, mammalian cells, plants and animal models by tissue‐specific expression of Cre recombinase 1, 2. More recently, conditional gene targeting using the Cre–*lox*P system has emerged as a powerful method in reproductive genetics and development biology, particularly in the study of embryonic lethal genes. Mouse lines expressing Cre recombinase under the control of different promoter regions are widely used in the study of mouse embryology and molecular genetics 3. These mice show great promise for tissue‐specific gene deletion and for contributing to the diagnosis and treatment of human diseases 4, 5.

*VASA*, also known as *DDX4*, is a gene that plays an important role in germ cell formation, spermato‐genesis, RNA splicing and cell growth. It encodes a member of the DEAD‐box family of ATP‐dependent RNA helicases, which is involved in regulation of mRNA translation in germ‐line differentiation 6, 7. Previous studies have demonstrated that *VASA* also plays roles in the establishment of the germ line in *Xenopus* frogs 8, zebrafish 9, 10, mice 11, humans 12, chickens 13 and rainbow trout 14. In addition, the *VASA* promoter region has been widely and effectively used as a germ cell marker or in germ cell‐specific transgenic zebrafish 9, 10, pigs 15, rainbow trout 16, mice 11 and chickens 13.

Although many Cre–*lox*P mouse models have been established, there are few pig models that take advantage of the Cre–*lox*P system. Pigs are thought to be the perfect nonhuman source of organs for xenotransplantation and are widely used as a disease model 17. In order to obtain a transgenic (Tg) pig line with germ cell‐specific expression of Cre, *VASA*–Cre Tg pigs with the Cre recombinase under the control of a 4320 bp 5′‐regulatory sequences of the porcine *VASA* were generated by somatic cell nuclear transfer (SCNT). We confirmed germ cell‐specific expression of Cre recombinase in *VASA*‐Cre Tg pigs. This will be a useful tool for germ line‐specific gene knockout and for use in disease models of reproductive system disorder.

## Materials and methods

### Ethics statement

All animal studies were conducted according to the experimental practices and standards approved by the Animal Welfare and Research Ethics Committee at Jilin University.

### Construction of *VASA*‐tdTOMATO and *VASA*‐Cre vectors

**Figure 1:**
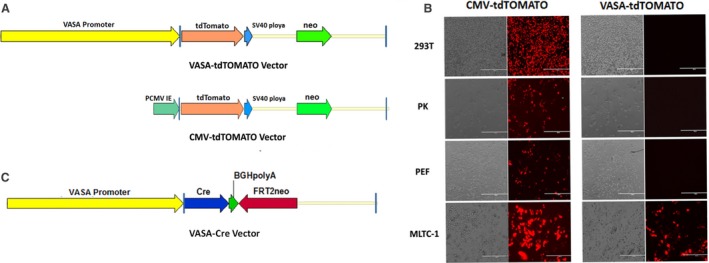
Specificity analysis of *VASA* promoter *in vitro*. (A) Construction of the *VASA*‐tdTOMATO vector. The 4.3 kb *VASA* promoter fragment was cloned into the vector of tdTOMATO. (B) Analysis of the expression of tdTOMATO in 293T, PK, PEF and MLTC‐1 cell lines. The CMV‐tdTOMATO vector was used as the positive control. (C) Construction of *VASA*‐Cre expression vector. The expression of Cre was controlled by the 4.3‐kb fragment of the pig *VASA* 5′‐flanking region, which was used to perform the SCNT in pig.

The 4320 bp 5′‐regulatory sequence of *VASA* (gene ID: 431 672) was PCR amplified from Landrace pigs' genomic DNA, which was cut with *Nhe*I and *Pci*I and cloned into the backbone of CMV‐tdTOMATO vector; the sequence was then confirmed (Fig. 1A). The forward and reverse primers of *VASA* are listed in Table S1. To test the specificity of the *VASA* promoter *in vitro*, the *VASA*‐tdTOMATO and the CMV‐tdTOMATO plasmids (positive control) were transiently transfected into cells of a pig kidney epithelial cell line (PK), human kidney epithelial cell line (293T), porcine fetal fibroblast cell line (PEF) and mouse Leydig tumour cell line (MLTC‐1), and the fluorescence intensity was determined with a fluorescence microscope (Nikon TS100, Tokyo, Japan).

For the construction of *VASA‐*Cre vectors, the 4320 bp fragment of the *VASA* 5′‐regulatory sequences was inserted into the *Nhe*I and *Sca*I sites of the pET28a‐Cre plasmid and the sequence confirmed 18. The expression of Cre was under the control of the pig *VASA* 5′‐regulatory sequences (Fig. 1C).

### Generation and identification of *VASA*‐Cre Tg pigs

The liberalized *VASA*‐Cre plasmid was transfected into Landrace‐ and mini‐pig‐derived foetal fibroblast cells using the FugeneHD reagent (Roche, Basel, Switzerland). After 24 h, the cells were split 1 : 36, and cultured in selection medium containing 400 μg·mL G418 (Amresco, Solon, OH, USA) for 10 days. Cell colonies were isolated, and incorporation of the plasmid was verified by PCR; the primer is listed in Table S1. Cells carrying the plasmid were selected as donor cells for SCNT, which has been described previously 19.

To identify the Tg pigs, the genomic DNA was isolated from tail tissue of newborn cloned pigs using the TIANamp Genomic DNA Kit (Tiangen Biotech, Beijing, China), and PCR was then performed using Cre‐F and Cre‐R primers (Table S1). Total RNA was isolated using the TRNzol reagent (Tiangen Biotech) according to the manufacturer's instructions. RNA was first treated with DNase I (Fermentas, Ottawa, Canada) and reverse transcribed to cDNA using the BioRT cDNA first strand synthesis kit (Bioer Technology, Hangzhou, China). *GAPDH* was used as an internal control using the primers *GAPDH*‐F and *GAPDH*‐R in Table S1.

### Western blot and immunohistochemical analysis

For western blotting, the tissue samples of cloned pigs were homogenized in 150 μL lysis buffer and protein concentrations were measured using the BCA Protein Assay Kit (Beyotime, Haimen, China). Goat anti‐Cre recombinase polyclonal antibody (1 : 1000; Santa Cruz Biotechnology, Dallas, TX, USA) was used to detect the expression of the Cre recombinase protein, and anti‐GAPDH monoclonal antibody (1 : 2000; Beyotime) was used as an internal control.

Immunohistochemistry (IHC) was performed as described previously 18. Briefly, testis of the Tg and wild‐type (WT) pigs were fixed in 4% paraformaldehyde, washed with 1× PBS and embedded in paraffin wax after 24 h The paraffin wax sections were pretreated with citrate buffer (0.01 m, pH 6.0) and blocked with normal goat serum. Primary antibodies were incubated on the slide at 4°C overnight, the slides were washed in 1× PBS, then incubated with donkey anti‐(goat IgG) antibody (1 : 500; Bioss, Beijing, China) for 20 min at room temperature. Finally, 2,4‐diaminobutyric acid (DAB) was used to label the IHC, and the sections were analysed under the microscope (Nikon TS100).

## Results

### Specificity analysis of *VASA* promoter *in vitro*

To determine the specificity of *VASA* promoter *in vitro*, the *VASA*‐tdTOMATO vector was transiently transfected into the somatic cell lines 293T, PK and PEF, and the germ cell line MLTC‐1; the CMV‐tdTOMATO vector was used as a positive control. Fluorescence microscopy was used to detect the expression levels of tdTOMATO (red fluorescent) in the transfected cells. The result showed that red fluorescence was readily observed in MLTC‐1 after 48 h, while not detected in the somatic cell lines 293T, PK and PEF (Fig. 1B), suggesting that the 4320 bp 5′‐regulatory sequences of *VASA* could be used to induce gene expression specifically in germ cells.

### Generation and identification of *VASA*‐Cre Tg mini‐pigs

**Figure 2:**
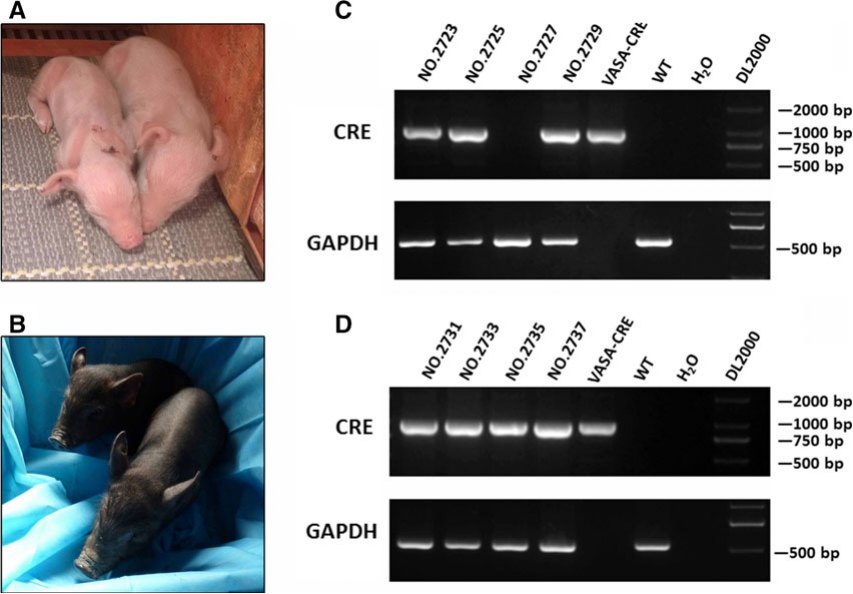
Generation and identification of *VASA*‐Cre Tg pigs. (A, B) Two of four surviving founder Landrace (ID nos 2723 and 2725) (A) and two surviving founder mini‐pigs (ID nos 2735 and 2737) (B). (C, D) PCR identification of the Cre gene in Tg Landrace (C) and mini‐pigs (D). All of the Tg pigs except No. 2727 were positive. *VASA*‐Cre vector served as postive control and *GAPDH* was used as the internal control in PCR analysis; a wild‐type piglet genomic sample (WT) and distilled water served as negative control in PCR analysis.

A total of 2842 reconstructed embryos were transferred into 10 recipient pigs (Table 1). Six recipients aborted during pregnancy and the other four produced eight male pigs, including four Landrace (Fig. 2A) and four mini‐pigs (Fig. 2B). Two mini‐pigs died 4 days after birth (ID No. 2731 and 2733, Table 1). The genomic PCR results showed that all of the cloned pigs, except No. 2727, were positive for the construct, showing a clear band of the Cre expression‐cassette in both cloned Landrace and mini‐piglets (Fig. 2C,D).

**Table 1:** Statistics of embryo transfer, pregnancy, and newborn cloned piglets

Donor cells	Recipient's ID no.	Embryos transferred	Number of piglets born	Piglet ID no.
Landrace	06	250	2	2723, 2725
	52	208	0 (aborted)	
	56	210	0 (no pregnancy)	
	57	245	2	2727, 2729
	63	220	0 (aborted)	
Mini pig	61	340	2	2731, 2733
	68	216	0 (no pregnancy)	
	70	220	2	2735, 2737
	94	220	0 (no pregnancy)	
	88	202	0 (aborted)	

### Specificity of *VASA*‐Cre expression in Tg pigs

**Figure 3:**
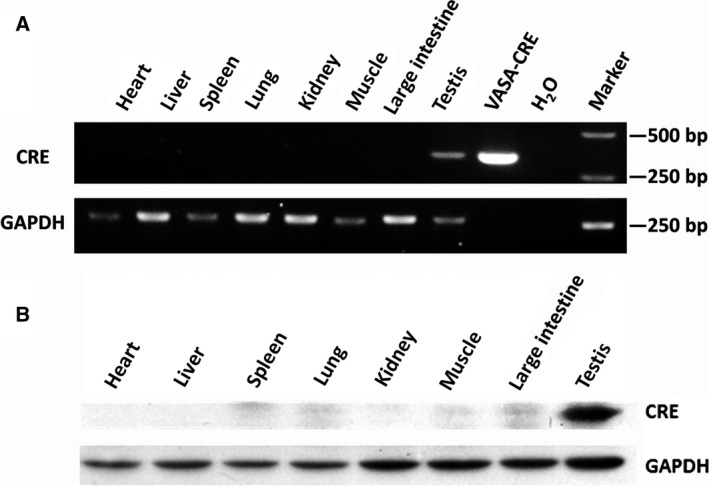
Specificity analysis of *VASA* promoter in Tg pigs. RT‐PCR (A) and western blotting (B) analysis of different tissues from *VASA*‐Cre Tg and WT pigs. *VASA*‐Cre vector served as a positive control and *GAPDH* was used as the internal control in RT‐PCR analysis.

To further test the specificity of the Cre expression in the *VASA*‐Cre Tg pigs, the Cre expression pattern of Tg pigs was analysed by RT‐PCR and western blotting. The RT‐PCR result showed that the Cre mRNA was specifically expressed in testis tissue of Tg pig, but not in other tissues of the Tg and WT pigs (Fig. 3A). This result was confirmed by western blot analysis (Fig. 3B), which demonstrated the Cre recombinase under the control of the 5′‐regulatory sequences of *VASA* was exclusively expressed in testis of Tg pigs.

### Analysis of Cre expression at the cellular level

**Figure 4:**
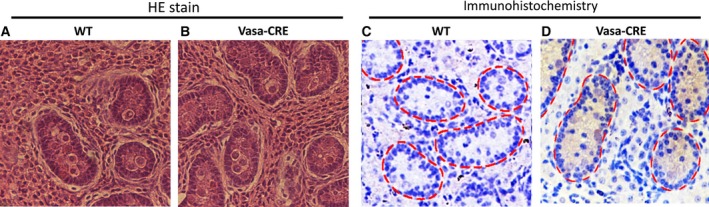
Cellular expression of Cre recombinase in testis of Tg pigs. Testis from Tg and WT pigs were analysed by HE staining (A, B) and IHC (C, D). Brown signals showed the expression of Cre recombinase and nuclei were stained with Hoechst. Cre was detected in the spermatogonia of the seminiferous tubules (red dashed line).

To determine if Cre expression was germ cell specific in Tg pigs, the haematoxylin–eosin (HE) staining and IHC analysis were performed on testis of Tg (No. 2731) and WT pigs. The HE result demonstrated that there is no significant histological difference between the testis of WT and Tg pig (Fig. 4A,B). Cre expression was observed in germ cells of the Tg pigs, but not in the germ cells of WT pigs, or somatic cells of Tg pigs in IHC analysis (Fig. 4C,D). These results suggest that the *VASA*‐Cre Tg pigs specifically express Cre recombinase in germ cells, and that the expression of Cre did not disrupt the development of testis in Tg pig.

## Discussion

As *VASA* is specifically expressed in germ cells in most species 11, 15, the analysis of its promoter could contribute to increase knowledge about its function in the future 20, 21, 22. In this study, a 4.3‐kb pig *VASA* promoter was used to construct *VASA*‐Cre Tg pigs. The length of the promoter region is similar to previous studies, which have demonstrated that the effective *VASA* 5′‐regulatory sequences was 5.1, 4.7, 2.7, 5.6 and 8 kb in medaka fish 23, rainbow trout 16, chicken 13, mice 11, and cows 22 respectively. In addition, although the longer promoter sequence can increase the specifically of the promoter, it also increased the difficulty of vector construction and the possibility of nonspecific gene expression. However, a 40 bp core promoter from positions −96 to −57 bp is necessary and sufficient to direct germ line‐specific gene expression in *Drosophila* 20, 21. In future studies, we will further investigate the core promoter region of *VASA* in pigs.

Although previously studies revealed that *VASA* is specifically expressed in germ cells of pigs 15, the specificity of the *VASA* 5′‐flanking promoter region has not been determined. We therefore performed an *in vitro* expression analysis of the *VASA* 5′‐regulatory sequence in the MLTC‐1 Leydig testis cell line before performing SCNT. We also tried to inject the *VASA*‐tdTOMATO plasmid into the porcine MII pronucleus, but the transgenic efficiency is very low (data not shown). Alternatively, we can use an *in vitro* transcript mRNA to improve the transgenic efficiency of the MII pronucleus in future studies.

Previous research has shown that *VASA* is germ cell lineage specific in invertebrates and vertebrates, and it has also been used as a marker for germ cells or germ cell‐specific Tg animals 11, 15. In this study, in order to verify the expression of *VASA* promoter‐driven Tg Cre pigs, we performed HE and IHC analyses. Previous reports showed germ cell‐specific LacZ expression in *VASA*‐Cre transgene mice 11, which was confirmed by our study in pigs. In addition, the testis tubules had not fully matured at 4 days in Tg testis, so the morphology and Cre expression of adult testis should be determined in future studies.

In conclusion, this is the first report of a germ cell‐specific Cre expression in mini‐pig and Landrace pigs. The efficiency and specificity of this *VASA*‐Cre Tg pig line demonstrated that it will be a useful tool for germ cell‐specific gene knockout and contribute to the functional analysis of genes in germ cells and in gonadogenesis and gametogenesis.

## Author contribution

LZJ and LLX conceived and designed the study. LL, HYY and WAF performed the experiments. TXC provided the mutants. SYN and LZJ wrote the paper. SYN and LZJ reviewed and edited the manuscript. All authors read and approved the manuscript.

## Supporting information


**Table S1.** Primers used in PCR or RT‐PCR.
