# Supplementary material for: Germ cell‐specific expression of Cre recombinase using the VASA promoter in the pig
Source: FEBS Open Bio. 2015 Dec 29;6(1):50–5. doi: 10.1002/2211-5463.12005 (PMC4794798; doi:10.1002/2211-5463.12005)
Supplement: Supplementary file 1 — Table S1. Primers used in PCR or RT‐PCR. [file FEB4-6-50-s001.docx]

**Table S1: Primers used in PCR or RT-PCR .**

| Name | Primers | Sequence(5’-3’) | Produce size(bp) |
| --- | --- | --- | --- |
| VASA | VASA-F | GGCTAGCCACAGATTTCAAGAGAGAAAGAAATGAGG | 4310 |
|  | VASA-R | GAGTACTCCCGTTCTTCATTTGACCCAAAGTCCACC |  |
|  |  |  |  |
| VASA-T | VASA-T-F | GACATGTCTTGTATGGTTAGTAAATGTAC | 4310 |
|  | VASA-T-R | GGCTAGCC CCGTTCTTCATTTGACCCAAAG |  |
|  |  |  |  |
| GAPDH | GAPDH-F | CACAGTCAAGGCGGAGAACGGGAA | 513(PCR)  266(RT-PCR) |
|  | GAPDH-R | CGATCTTGAGGGAGTTGTCATAC |  |
| CRE-1 | CRE-1-F | GAATTCTCAATCGCCATCTTCCAGC | 944 |
|  | CRE-1-R | AAGCTTATGGCCAATCTCCTGACCG |  |
|  |  |  |  |
| CRE-2 | CRE-2-F | CCACGCGCCCTGGAAGGGA | 354 |
|  | CRE-2-R | CATGTCTCTTGCGGCTCCGA |  |
